# The Veterinary Department of the University of California

**Published:** 1896-07

**Authors:** 


The Veterinary Department of the University of California, not having any students who had spent the prescribed term of three years, closed its session without any exercises. Some fourteen students were in attendance at the several courses during the past year.

